# Care of patients with Phenylketonuria (PKU) in Germany – a claims data analysis from 2013 to 2023

**DOI:** 10.1186/s13023-026-04467-3

**Published:** 2026-07-02

**Authors:** Axel Boehnke, Lisa-Marie Müller, Constantin Heidecke, Alexa Benson, Aljoscha S. Neubauer, Ania C. Muntau

**Affiliations:** 1 PTC Therapeutics Germany GmbH, The SQUAIRE 12, Am Flughafen, 60549 Frankfurt am Main, Germany; 2 Gesundheitsforen Leipzig GmbH, Hainstr. 16, 04109 Leipzig, Germany; 3 IfGPh Institut für Gesundheits- und Pharmakoökonomie GmbH, Frau-Holle-Str. 9A, 81739 Muenchen, Germany; 4 University Children’s Hospital, University Medical Center Hamburg-Eppendorf and German Center of Child and Adolescent Health (DZKJ), Martinistraße 25, 20246 Hamburg, Germany

**Keywords:** Phenylketonuria (PKU), Rare disease, HCRU, Real-world data, Comorbidities, Pharmacological treatment, Rehabilitation care, Maternal PKU syndrome

## Abstract

**Background:**

Phenylketonuria (PKU) is a rare inherited metabolic disorder requiring lifelong management to prevent neurological, psychiatric, and physical complications. Although early detection through newborn screening has improved outcomes, real-world evidence on long-term care and healthcare utilisation remains limited. This study assessed the epidemiology, comorbidities, treatment practices, rehabilitation and healthcare resource utilisation (HCRU) of individuals with PKU in Germany, using nationwide statutory health insurance (SHI) claims data over ten years.

**Results:**

Based on anonymised claims data of 16 SHI funds (~ 4.4 million insured; 5% representative sample), 4,949–6,105 PKU patients were identified annually from 2013 to 2023, yielding a prevalence of 0.007%–0.008%. The five-year cumulative prevalence (2019–2023) was 0.010%, with a slight female predominance (52%). Compared to matched controls (1:10), PKU patients had higher odds of comorbidities, including intellectual disability (OR 16.0), other metabolic disorders (OR 9.2), osteoporosis (OR 3.4), hyperkinetic disorders (OR 2.1), and recurrent depressive disorders (OR 1.6), with the severity of cognitive impairment increasing with age. Less than 20% of the individuals received pharmacological treatment, with low incidences in patients older than 54 years. Nutritional therapy was common during childhood, adolescence and young adulthood (average 49% in ages 0–24) but declined with increasing age. Pregnancy terminations were documented in more than 15% of the 34 women with recorded pregnancy in the cohort. Rehabilitation care was overall higher than in the healthy population, especially among children aged 2–12 years (5.6% vs. 0.3% in controls), primarily for developmental disorders. Healthcare costs were considerably higher in the PKU cohort.

**Conclusions:**

PKU care in Germany exhibits age-dependent differences in documented treatment patterns and a high burden of comorbidities, highlighting a substantial unmet medical need and the importance of structured lifelong management strategies.

**Supplementary Information:**

The online version contains supplementary material available at 10.1186/s13023-026-04467-3.

## Background

Phenylketonuria (PKU) is a rare inherited metabolic disorder caused by a defect in phenylalanine hydroxylase (PAH), leading to elevated phenylalanine (Phe) and reduced tyrosine levels [1, 2]. Without targeted management, PKU can result in profound developmental, neurological, and psychiatric impairments [1] and may give rise to a broader spectrum of neuropsychiatric and somatic comorbidities [3].

Since the introduction of newborn screening in Germany in 1969 [4], treatment is typically initiated within the first few days of life, which is crucial for preserving neurocognitive development, especially during infancy and childhood [2, 5]. Management centres on a low Phe diet supplemented with Phe free amino acid formulas and medical foods [3]. Despite structured dietary counselling and psychosocial support at metabolic centres, long-term adherence becomes increasingly difficult in adulthood, and dietary therapy alone often does not allow for optimal Phe control [6–8].

The use of pharmacological compounds has expanded the treatment options. Since 2009, sapropterin dihydrochloride, a synthetic form of the PAH cofactor BH₄, has been approved in Europe [9]. It enhances residual enzyme activity in some individuals, although only 20–50% of patients respond. Residual enzyme activity is a prerequisite for treatment response [10]. For patients with the severe form of the disease lacking residual enzyme activity, pegvaliase, approved in Europe since 2019 for patients aged ≥ 16 years, serves as an enzyme substitution therapy that operates independently of PAH and effectively lowers blood Phe, although efficacy may be delayed until immune tolerance is established, which can take from several weeks to more than a year, and is frequently accompanied by immunogenic adverse events during the early phase of treatment [11–13].

In addition to neuropsychological concerns, adults with PKU frequently have reduced bone mineral density despite sufficient vitamin D and calcium [2, 14], mineral deficiencies, obesity, especially in women and those with classical PKU, and more frequent cardiometabolic, gastrointestinal, respiratory, and musculoskeletal comorbidities [3, 15–19]. Although lifelong treatment is recommended, it imposes a significant burden on patients [2].

Pregnancy introduces a critical risk in women with PKU, as elevated maternal Phe levels are teratogenic and can lead to severe foetal malformations (maternal PKU syndrome (MPKUS)), necessitating strict dietary control starting before conception [20]. Women with PKU often experience cognitive and emotional stress during pregnancy, underscoring the need for focused education and support as the number of pregnancies in this population increases [20–22].

The burden of PKU extends beyond the individual and can only be fully understood when considering the various healthcare sectors and the full spectrum of medical services utilised by affected patients, as reflected in the significantly higher mean healthcare costs for individuals with PKU [23]. In line with this, recent evidence indicates a substantial economic impact, with individuals with PKU incurring higher healthcare costs than unaffected controls [24]. Among early diagnosed patients, increased expenditures were mainly driven by outpatient pharmaceutical costs, particularly for medical food and amino acid supplements. In contrast, for patients diagnosed later in life, inpatient care accounted for most excess costs. In addition to these direct medical costs, global evidence highlights a considerable indirect cost burden, including productivity losses, caregiver time, and other non-medical expenses, which further amplify the overall societal impact of PKU [25–27]. Beyond financial aspects, individuals with PKU also face limitations in social participation, report feelings of isolation, and encounter barriers to education and employment [28].

In light of these challenges, this study aimed to provide a comprehensive real-world overview of the care of patients with PKU in Germany. The goal is to generate evidence-based insights into care patterns, treatment practices, and existing gaps to inform future health policies and clinical management.

## Methods

### Data source

This study used health claims data from a German statutory health insurance (SHI) database (“Deutsche Analysedatenbank für Evaluation und Versorgungsforschung” [DADB]), which is administered by Gesundheitsforen Leipzig GmbH. The DADB comprises anonymised, routinely collected claims data from 16 German sick funds between 2013 and 2023. The dataset includes approximately 4.4 million insured individuals, corresponding to approximately 5% of the population covered by SHI in Germany, which accounts for approximately 90% of the total population [29]. The DADB includes insured individuals from multiple sick funds that are generally open nationwide and has previously been evaluated regarding its representativeness for the German SHI population. While the unadjusted database shows a slightly younger population structure compared to the overall SHI population, age-, sex-, and risk-adjustment approaches based on the German morbidity-based risk adjustment scheme (Morbi-RSA) substantially improve representativeness regarding demographic and morbidity characteristics.

### Study design and patient selection

This retrospective, non-interventional cohort study included all individuals with at least one year of comprehensive insurance coverage during the study period from 1 January 2013 to 31 December 2023. Patients who died were not excluded. Patients were considered prevalent coded with PKU if they had at least one confirmed outpatient or inpatient diagnosis of PKU within the year. Diagnoses were identified using the ICD-10 codes E70.0 (classical phenylketonuria) and E70.1 (other hyperphenylalaninemias). Both codes were included deliberately, as the study aimed to capture the spectrum of patients relevant to PKU-related healthcare provision, including milder phenotypic manifestations. This approach is consistent with clinical classification systems such as Orphanet, which reference both ICD-10 codes within the PKU spectrum [30]. In addition, ICD-based routine data do not provide sufficient granularity regarding disease severity or detailed phenotypic manifestations within hyperphenylalaninemia disorders.

Additional analyses used outpatient procedure reimbursement codes (German EBM) and the WHO Anatomical Therapeutic Chemical classification system (ATC codes) for drugs. For the HCRU analyses, individuals were defined as incident if they received a diagnosis in the reporting year but not in the baseline year. HCRU in this study was defined as the use of reimbursed healthcare services captured in claims data. This measure reflects the extent to which patients engage with the healthcare system across all relevant service sectors. Our analysis focused exclusively on direct medical service utilisation. Indirect aspects and costs, including productivity losses, caregiver time, or other non-medical impacts, were not considered, as quantifying the full societal cost of PKU was not the objective of this study.

### Age groups

For this analysis, age groups were defined in advance based on considerations specific to PKU, including relevant developmental stages, transitions in disease manifestation, care needs, and treatment practices. The age groups were as follows: 0–1 year (infants), 2–12 years (children), 13–15 years (early adolescents), 16–17 years (late adolescents), 18–24 years (young adults), 25–54 years (adults), 55–64 years (older adults), and ≥ 65 years (elderly). Differentiation into young adults and adults aimed to consider the time needed for higher education before entering the labor market entry. Individuals born before the introduction of systemised newborn screening for PKU were covered in the age group of > 55 years.

### Matching and odds ratios

For analyses requiring a comparison group, a 1:10 matched control cohort was created for each patient with a confirmed PKU diagnosis. Controls were matched according to the calendar year, age, sex, and number of available follow-up years. Furthermore, for the analysis of comorbidities, matching also considered the prevalence of type 1 diabetes (ICD-10: E10). Type 1 diabetes was selected as an additional matching variable because it represents a chronic condition that, similar to PKU, requires long-term disease management, dietary considerations, and regular blood monitoring, and may therefore be associated with increased healthcare contact and diagnosis intensity [31]. Given the chronic nature of PKU, individuals were only eligible as controls if no diagnosis of PKU (ICD-10: E70.0 or E70.1) was recorded in the index year or the year preceding it. Therefore, analyses that include the matched cohort are available only for the years 2014 to 2023. Comparative analyses included the calculation of odds ratios (ORs) with corresponding 95% confidence intervals (CIs) and p-values using conditional logistic regression, stratified by year, age, and sex. Where applicable, extrapolated estimates were additionally reported with 95% confidence intervals to reflect statistical uncertainty, particularly in the context of rare disease analyses and small subgroup sizes. All analyses were exploratory, and no corrections for multiple comparisons were applied.

### Outcomes

Where applicable, results derived from the DADB were age- and sex-adjusted and extrapolated (abbreviated: ext.) to the SHI population in Germany using established weighting approaches. To quantify the statistical uncertainty, 95% CIs around the annual case counts were calculated using stratified Wilson score intervals [32].

Patients were eligible for inclusion in multiple annual cohorts if they satisfied the diagnostic criteria for more than one year. For specific analyses, a cumulatively prevalent population encompassing the entire study period (2013–2023) was included. Depending on the research question, a patient could be counted once or multiple times within an age group. Extrapolation to the SHI population was conducted using the adjustment factor from the earliest year in which the respective individual met the definition of prevalence. As individuals could transition into new age groups over time, they might contribute to more than one age group. This is also applicable to the analysis of cumulative PKU prevalence, utilising data collected over a five-year period (2019–2023). Individuals were considered prevalent if they had received at least one confirmed diagnosis of PKU within the specified timeframe.

In compliance with data protection regulations, results derived from fewer than ten individuals are denoted as “n < 10.” Consequently, subgroups comprising fewer than ten cases could not be further disaggregated or visualised. The threshold of *n* < 10 pertains to the sample population within the DADB. An observed value of *n* = 0 signified that no cases were identified in the DADB sample for the respective group.

### Ethics approval and consent to participate

The study was conducted in accordance with established best practices for secondary claims data analyses, including the Good Practice of Secondary Data Analysis (GPS), the Standardized Reporting Of Secondary Data Analyses (STROSA 2) guideline, and the Strengthening the Reporting of Observational Studies in Epidemiology (STROBE) statement [33–35]. Ethical approval was not required because only fully anonymised sick funds claims data were used. Informed consent was not required because no identifiable personal information was available to the investigators.

## Results

### Prevalence

In the investigated time frame between 2013 and 2023, the cohort of continuously insured DADB population increased from 2,606,813 to 3,022,933 individuals. The number of individuals with a confirmed diagnosis of PKU within the DADB varied annually, ranging from 178 (ext.: 4,510 CI: 4,089; 5,484) to 248 (ext.: 6,105 CI: 5,466; 7,010) cases. The resulting prevalence rates ranged between 0.006% and 0.008%. The estimated prevalence in the SHI population corresponded to 6.25–8.09 cases per 100,000 insured individuals across the observation period. Across all study years, a slightly higher proportion of PKU cases was consistently observed among females, ranging from 51.2% to 57.6%.

The overall five-year cumulative prevalence in the SHI population was 0.010%. The calculated age-specific five-year prevalence estimates decreased with age: 0.026% for infants aged 0–1 year, 0.024% for children aged 2–12 years, 0.016% for adolescents aged 13–15 years, and 0.012% for those aged 16–17 years. The five-year prevalence further decreased to 0.010% in young adults aged 18–24 years, followed by 0.007% in adults aged 25–54 years, 0.003% in those aged 55–64 years, and 0.006% in those aged ≥ 65 years.

### Comorbidities

**Fig. 1:**
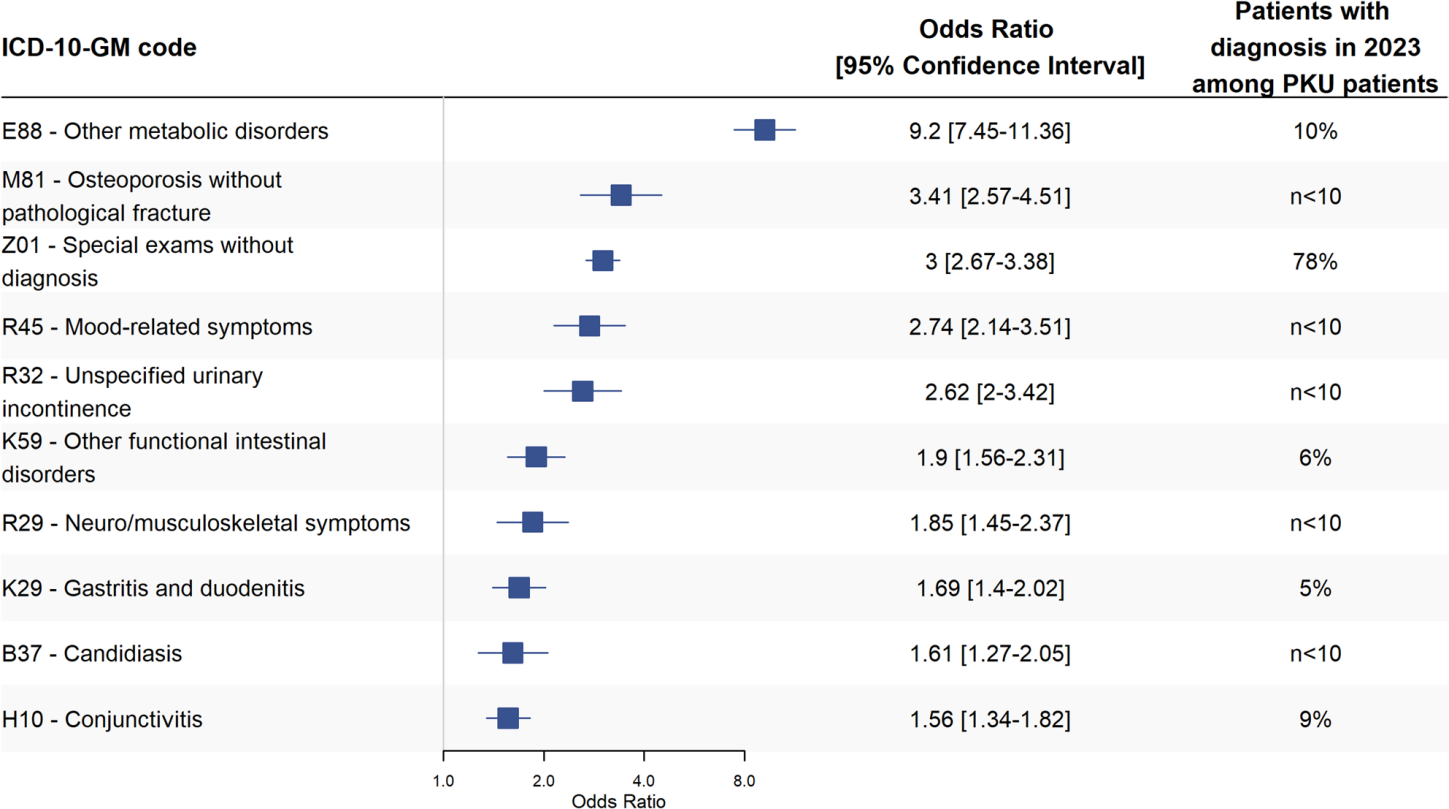
Most frequent comorbidities based on odds ratios in the PKU population compared to a matched cohort. Odds ratios [± 95% confidence interval] from the conditional logistic regression. Percentages are based on SHI extrapolated values

To evaluate the extent of multimorbidity, all disease diagnoses (by three-digit ICD-10) in addition to PKU were compared between patients with PKU and the matched control group. This level of aggregation was chosen to provide a structured overview of broader and clinically relevant comorbidity patterns while avoiding excessive fragmentation across highly specific diagnostic subcodes in the context of a rare disease. The analysis was therefore intended to identify broader comorbidity domains rather than highly specific diagnostic subentities. Both confirmed outpatient and inpatient diagnoses were considered. Compared with the matched control group, patients with PKU had significantly higher odds for several diseases (Fig. 1).

Not regarding mental and behavioural disorders, the strongest associations were observed for “other metabolic disorders” (ICD-10: E88, OR: 9.20, CI: 7.45; 11.36) and “osteoporosis without pathological fracture” (ICD-10: M81, OR: 3.41, CI: 2.57; 4.51). Moreover, “special exams without diagnosis” (ICD-10: Z01, OR: 3.00, CI: 2.67; 3.38) showed higher odds. Substantially elevated odds were also found for “mood-related symptoms” (ICD-10: R45, OR: 2.74, CI: 2.14; 3.51), “unspecified urinary incontinence” (ICD-10: R32, OR: 2.62, CI: 2.00; 3.42). All diagnoses that show a statistically significant odds ratio > 1 are reported in Table S1.

**Fig. 2:**
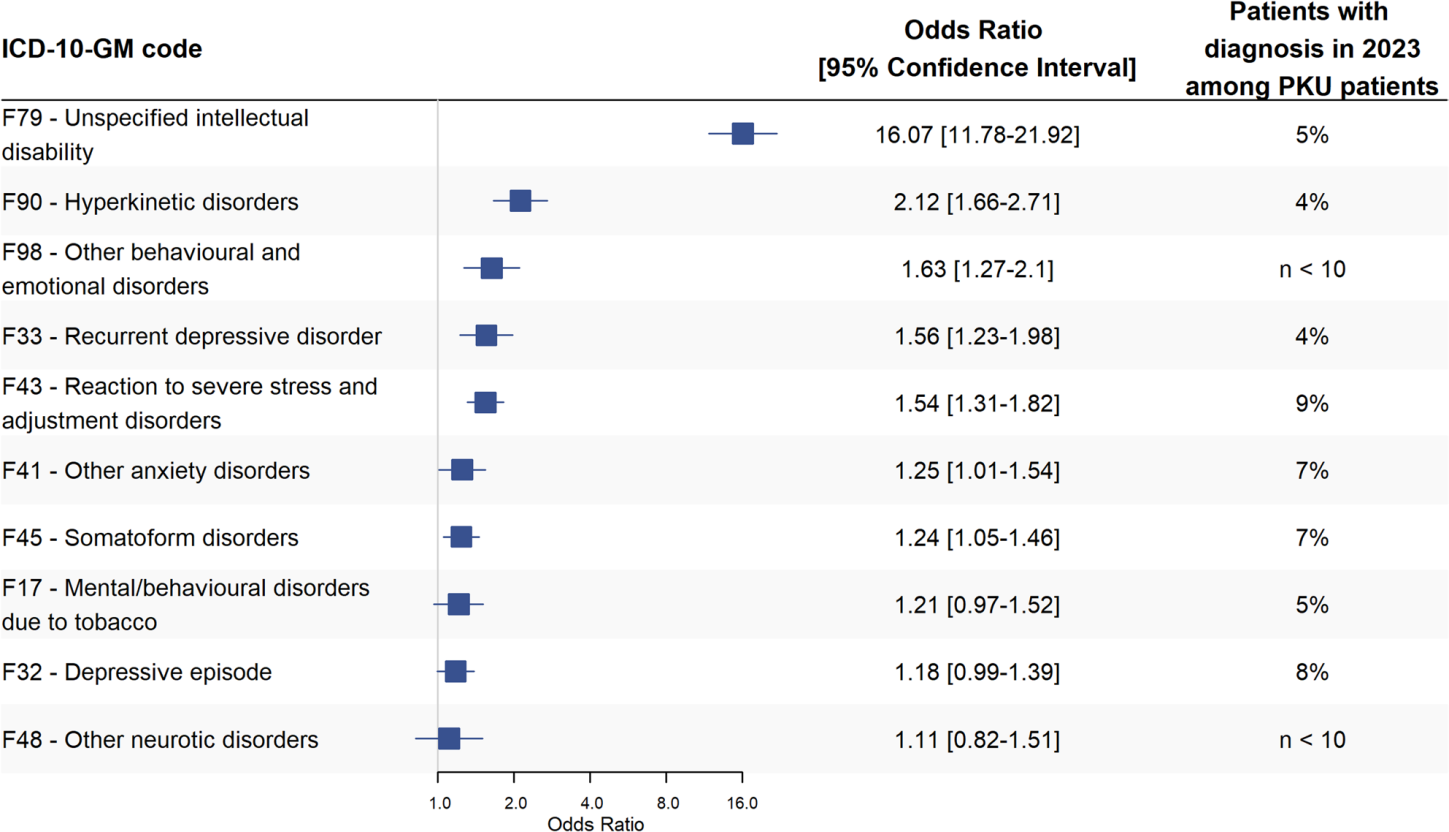
Most frequent F-diagnosis comorbidities based on odds ratios in the PKU population compared to a matched cohort. Odds ratios [± 95% confidence interval] from the conditional logistic regression. Percentages are based on SHI extrapolated values

Considering specifically the 10 most frequently observed mental and behavioural disorders (as per ICD-10), individuals with PKU showed markedly increased odds for several diseases compared to matched controls. The strongest association was observed for “unspecified intellectual disability” (ICD-10: F79, OR: 16.07, CI: 11.78; 21.92). Statistically significant associations were also found for “hyperkinetic disorders” (ICD-10: F90, OR: 2.12, CI: 1.66; 2.71), behavioural and emotional disorders with onset in childhood (ICD-10: F98, OR: 1.63, CI: 1.27; 2.10), “recurrent depressive disorder” (ICD-10: F33, OR: 1.56, CI: 1.23; 1.98), and adjustment disorders (ICD-10: F43, OR: 1.54, CI: 1.31; 1.82) (Fig. 2).

### Pregnancy

**Fig. 3:**
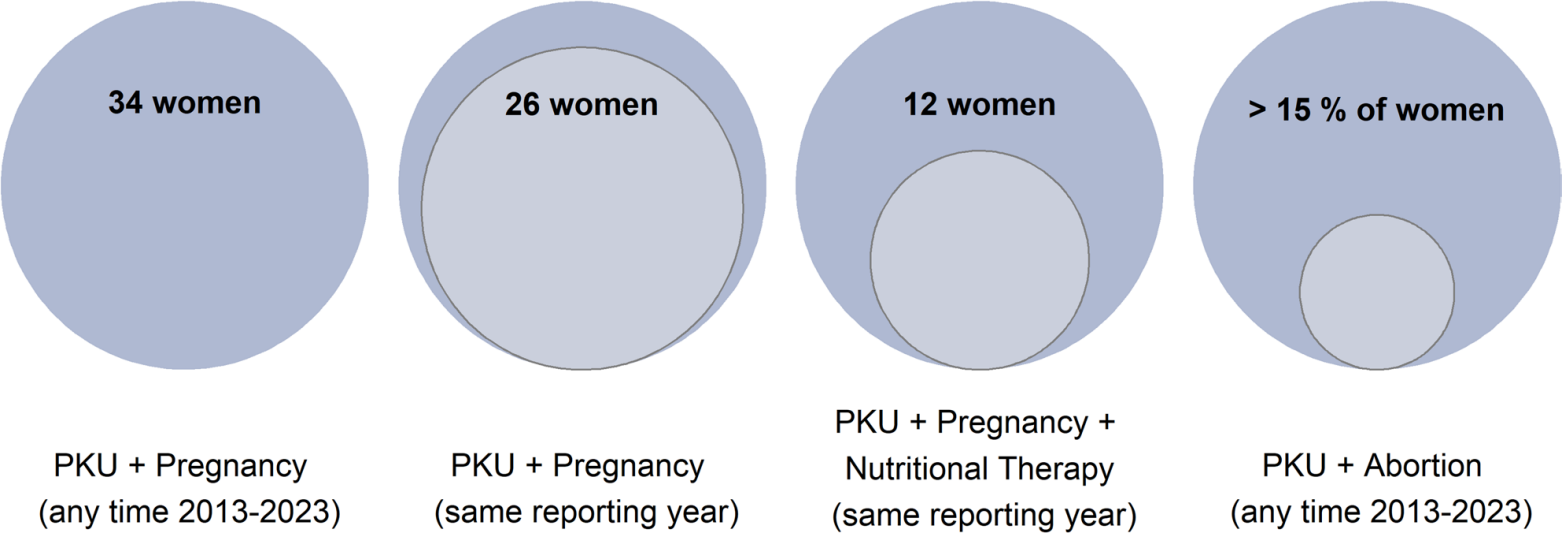
Visual representation of pregnancy-related care pathways among women with PKU. The diagram illustrates the number of women with confirmed PKU diagnoses who were identified as pregnant during the observation period (2013–2023; *n* = 34)

The management of pregnancy in women with PKU was also examined in this study. During the observation period, 34 (ext.: 808 CI: 577; 1,139) women with confirmed PKU diagnoses were identified in the DADB as being pregnant (Fig. 3). In 26 (ext.: 641 CI: 439; 943) cases (79%), both pregnancy and PKU diagnoses were recorded in the same year. Among these 26 cases, 12 (ext.: 298 CI: 171; 524) women (46%) received dietary therapy in the year of prevalent PKU. Of note, pregnancy terminations were documented in more than 15% of the 34 women with a recorded pregnancy during the observation period. For reference, in 2024, the national average was 6.2 per 1,000 women in Germany, which calculates less than one termination in the sample of 267 (ext.: 7,213 CI: 6,368; 8,191) women with PKU, approximately half of them in the age groups 16 to 54 years [36]. Due to the small number of observed cases, this finding should be interpreted descriptively and with caution.

### Pharmacological treatment

Less than 20% of patients with PKU received at least one prescription of pharmacological treatment (sapropterin dihydrochloride or pegvaliase) during the study period. The highest treatment rate was observed in the age group from 18 to 24 years. Fewer than ten patients in the dataset were treated with pegvaliase (ATC: A16AB19), which, following its approval, was prescribed exclusively to individuals aged 16 years and older. None of these patients switched to sapropterin dihydrochloride during follow-up. Within the DADB, sapropterin dihydrochloride (ATC: A16AX07) was initiated in 48 patients; of these, 21 (44%) experienced a reinitiation, defined as a gap of ≥ 90 days without medication coverage, and 11 (23%) discontinued treatment, defined by the absence of any prescriptions for at least one full calendar year.

The prescription frequencies of pegvaliase and sapropterin dihydrochloride varied across age groups. The highest average annual number of prescriptions per patient with prescriptions was observed in the youngest age group (0–1 year: 4.4 per year) and older adolescents (16–17 years: 4.7 per year). Lower prescription frequencies were observed in children aged 2–12 years (3.2 per year) and adults aged 25–54 years (3.3 per year). No pharmacological treatment was recorded in patients aged ≥ 55 years.

### Blood tests

**Fig. 4:**
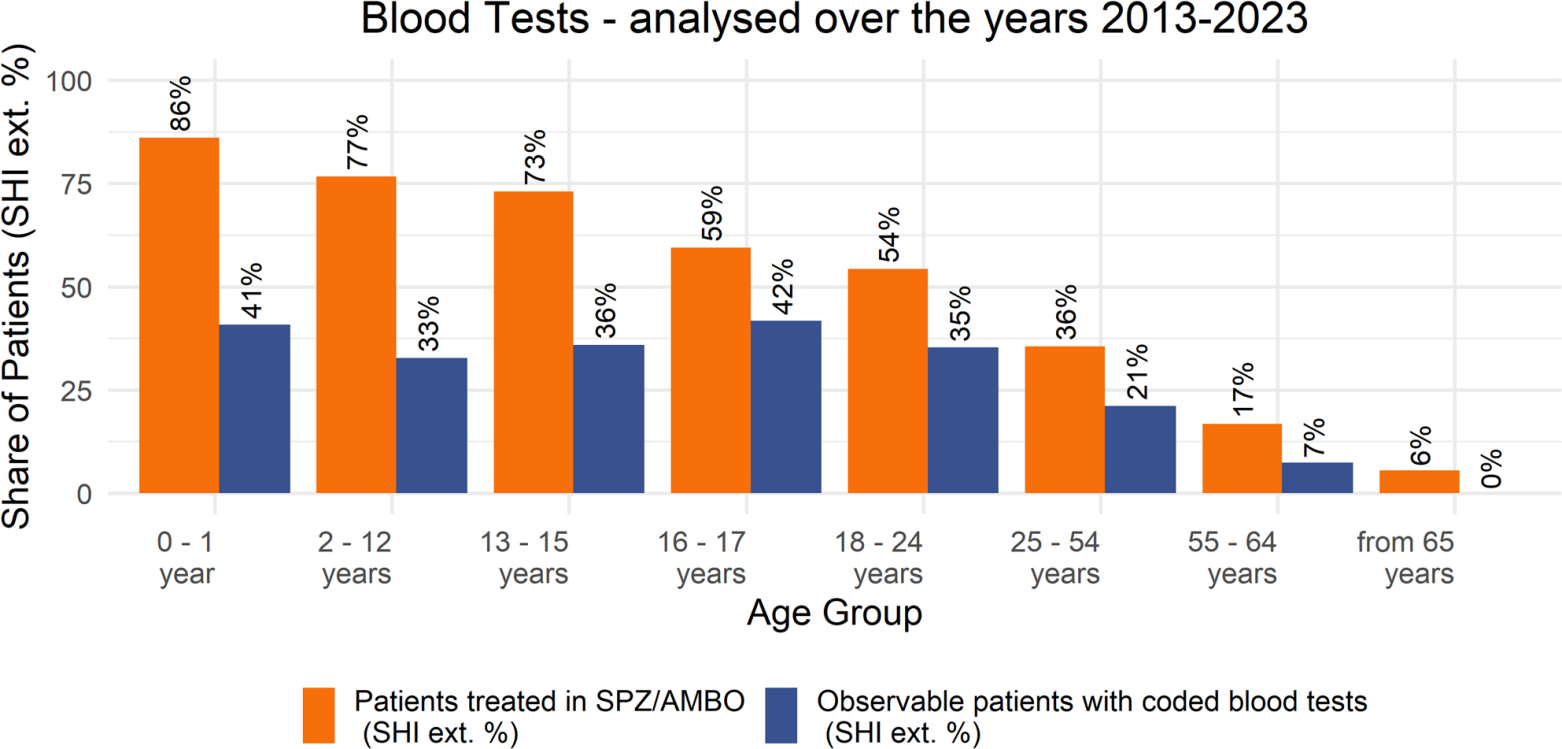
Age-stratified rates of Phe blood testing and care in SPZ/AMBO settings among PKU patients (2013–2023). The bar plot displays the extrapolated proportion of patients with documented blood tests for Phe monitoring (orange) and those treated in social paediatric centres (SPZ) or specialised outpatient clinics (AMBO; blue), stratified by age group

Throughout the observation period from 2013 to 2023, the annual rates of documented blood tests for Phe monitoring, as identified through relevant outpatient billing codes (EBM codes) for patients with PKU, varied between 21% in 2021 and 30% in 2016, with a rate of 25% recorded in the most recent year, 2023. Analysis of age group stratification across all reporting years indicated that the highest monitoring frequencies were observed among late adolescents, with a peak of 42% in the 16–17 years age group (Fig. 4). In younger cohorts, monitoring rates varied from 41% in infants (0–1 year) to 33% in children (2–12 years). Conversely, monitoring was less prevalent among adults aged 25–54 years, with 21%, and was only 7% in the 55–64 age group. No cases were documented in patients aged ≥ 65 years (*n* = 0).

An in-depth analysis of patients lacking documented Phe blood tests indicated that a large proportion of care was provided in social paediatric centres (SPZ) and specialised outpatient clinics (AMBO), where flatrate complex codes were employed. In these healthcare settings, specific services such as blood testing are frequently not coded separately and consequently remain undetectable in claims data. This structural limitation likely accounts for lower documentation rates, particularly among younger age groups. Most infants (0–1 year: 86%) and children (2–12 years: 77%) received care in SPZ/AMBO settings. The utilisation of these services remained substantial among early adolescents (13–15 years: 73%), older adolescents (16–17 years: 59%), and young adults (18–24 years: 54%), but decreased notably in adults aged 25–54 years (36%) and was lowest among individuals aged 55 years and older (17% or less).

### Nutritional therapy

**Fig. 5:**
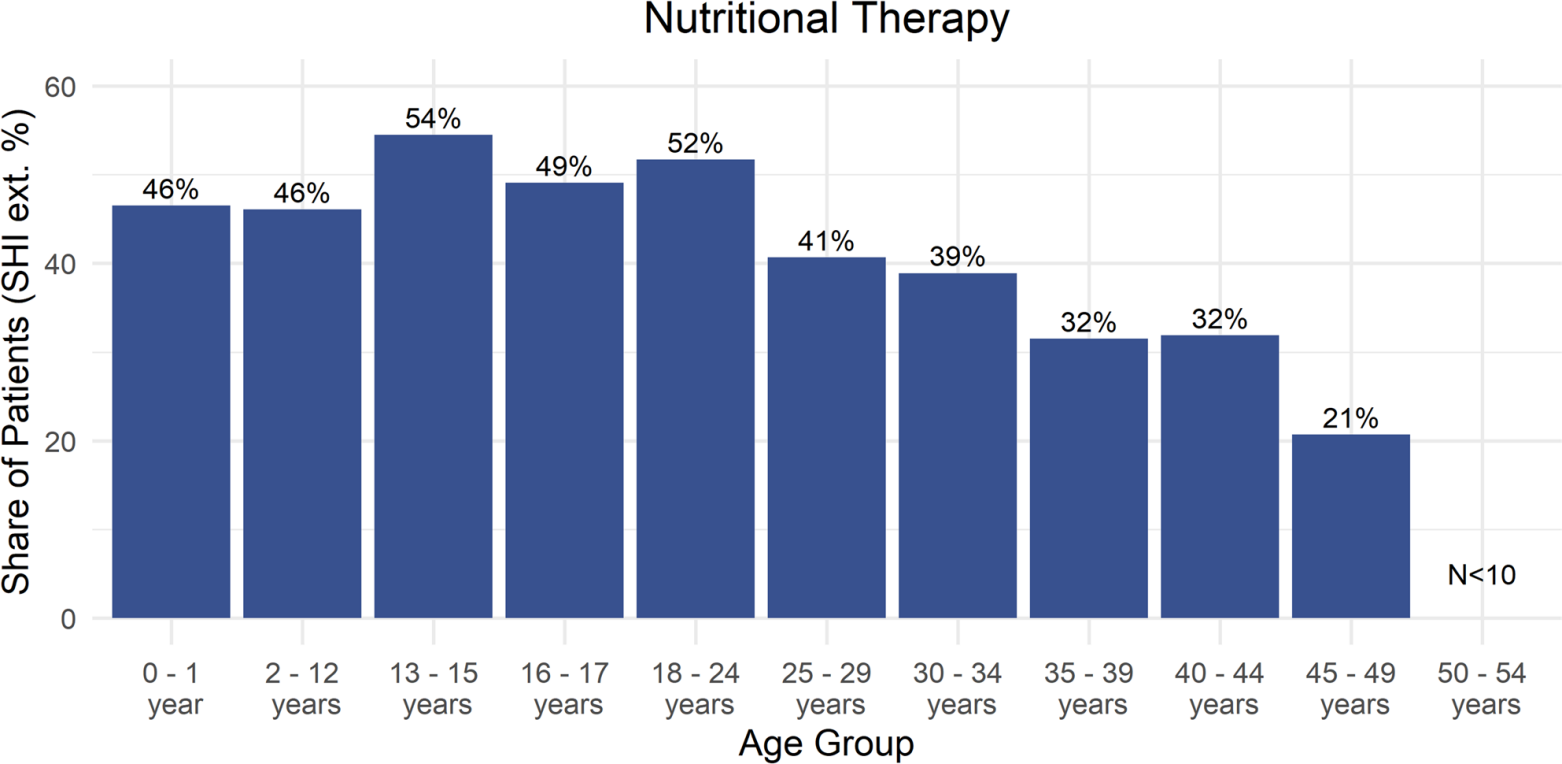
Age-stratified documentation of nutritional therapy among PKU patients across all years (2013–2023) (SHI extrapolated %). The proportion of patients with documented prescriptions for nutritional therapy (ATC code V06 or relevant EBM codes) across age groups is shown

Nutritional therapy was identified using documented prescriptions with the ATC code V06 and relevant EBM billing codes. Accordingly, the analysis reflects nutritional therapy observable in SHI claims data and may not capture all forms of dietary management provided in routine care. During the observation period, the proportion of patients with PKU receiving nutritional therapy increased from 31% in 2013 to 43% in 2023. This upward trend may reflect an increasing emphasis on dietary management. Age stratification revealed significantly higher utilisation among minors (average: 48%) than among adults (23%). The highest utilisation was observed in early adolescents aged 13–15 years (55%), followed by young adults aged 18–24 years (52%). A continuous decline was noted in older age groups: 41% (25–29 years), 39% (30–34 years), 32% (35–39 years), 32% (40–44 years), and 21% (45–49 years). Results for patients aged ≥ 50 years were not reported because of small sample sizes (*n* < 10). On average, nutritional therapy was documented in 48% of minors (< years) and 23% of adults (≥ 18 years) (Fig. 5).

### Healthcare resource utilisation

Annual total healthcare expenditures among incident patients with PKU showed substantial variation over time and consistently exceeded those observed in a matched control population (Table S2; Table S3). Total expenditures include all major healthcare service areas, such as physician services, dental care, pharmaceuticals, hospital treatments, other services, sickness benefits, extracorporeal blood purification, and discretionary benefits under the SHI. In the PKU cohort, mean annual costs ranged from €3,254 in 2016 to €20,485 in 2020, with other notable peaks in the years 2017 (€9,264), 2019 (€10,538), and 2023 (€7,067). In comparison, the mean costs in the control group were markedly lower across all years, ranging from €1,817 in 2017 to €3,247 in 2023, indicating a more stable cost pattern in the general population.

The median values further underscore the disparity: in the PKU population, the highest median annual cost was observed in 2020 (€7,580), followed by 2019 (€4,400) and 2022 (€2,380). By contrast, the lowest medians were observed in 2015 (€1,165), 2021 (€1,206), and 2016 (€1,595). For the control group, the median costs remained consistently below €1,000 in all years, ranging from €566 in 2020 to €825 in 2022.

The cost extremes also differed notably; the maximum cost in the PKU group reached €281,691 in 2020, followed by €104,770 in 2017 and €56,029 in 2023. In the control group, the maximum annual costs ranged from €27,904 in 2016 to €155,229 in 2023, with lower interindividual variation in most years.

Across all reporting years, the minimum annual total healthcare costs in the PKU cohort consistently exceeded zero, ranging from €31 (2020) to €340 (2023). This indicates that all individuals with PKU incurred at least some level of reimbursed healthcare expenditure each year. By contrast, the matched control group showed a minimum of €0 in every year except 2020, when the settlement of the billing data resulted in a negative value (–€354). This deviation is attributable to internal reimbursement processes within the SHI system, where inter-year accounting adjustments can occasionally result in negative cost entries.

### Hospital expenditures

Hospital-related expenditures were consistently higher in the incident PKU cohort than in the matched population across nearly all reporting years (Table S4; Table S5). The mean annual inpatient costs for patients with PKU ranged from €1,090 (2022) to €12,087 (2020), with notable peaks in 2017 (€5,361) and 2023 (€4,786). By contrast, the control group showed substantially lower values, with mean costs fluctuating between €674 (2016) and €1,873 (2023). The maximum annual inpatient costs observed among individuals with PKU reached up to €249,186 in 2020. This contrasts with the control population, where the maximum value was €150,315 (2023).

Across all age groups, individuals with PKU were more frequently hospitalised than their matched controls. The largest discrepancies were observed in infants (0–1 year): 46% of patients with PKU had at least one hospitalisation compared to 21% in the control group. Elevated rates were found in children aged 2–12 years (16% vs. 7%) and adolescents aged 13–17 years (13–15% vs. 7–8%). In adulthood, the hospitalisation proportion among patients with PKU remained above that observed in the control population, particularly in older adults. Among individuals aged 55–64 years, 23% of the PKU cohort had at least one hospital admission vs. 16% in the matched group; in those aged ≥ 65 years, the difference widened to 29% vs. 25%. The average number of hospitalisations per patient with at least one admission ranged from 1.1 (ages 16–17) to 1.8 (≥ 65 years) in the PKU group, and from 1.2 to 1.7 in the matched cohort. The mean lengths of stay were broadly similar between the groups, typically ranging between 4 and 9 days.

### Outpatient costs

Outpatient healthcare expenditures were consistently higher among individuals with incident PKU compared to the matched population across all years (Table S6; Table S7). Mean annual costs in the PKU cohort ranged from €745 (2016) to €1,819 (2020). Particularly elevated values were observed in 2017 (€1,159), 2018 (€1,075), 2019 (€1,226), and 2022 (€1,234). By contrast, the control group exhibited substantially lower mean costs, ranging between €458 (2014) and €591 (2023), with medians consistently below €400. Minimum costs were frequently €0 in the control population, indicating that a notable proportion of individuals had no billed outpatient services in a given year, which contrasts with the PKU group, where minimum values always exceeded €30. Maximum annual outpatient costs in the PKU cohort reached up to €17,201 (2020), while the highest value in the matched population was €7,089 (2017).

Drug costs were consistently and substantially higher in the incident PKU population compared to the matched control group across all years (Table S8; Table S9). The mean annual drug costs in the PKU cohort ranged from €676 in 2023 to a peak of €5,840 in 2020. By contrast, the corresponding values in the control group ranged only between €172 (2017) and €593 (2021), with most years remaining well below €500. The largest absolute differences in mean costs were observed in 2020 (€5,840 vs. €225), 2019 (€3,203 vs. €344), and 2017 (€1,987 vs. €172). The medians followed a similar trend, with notably higher values in the PKU group, ranging from €24 (2023) to €663 (2022). While some years showed moderate medians (e.g. €109 in 2018, €138 in 2019), the matched population remained consistently low across all years, with median pharmacy costs between €24 and €59. This suggests that the high average costs in the PKU group are not solely driven by outliers but reflect broader treatment-related needs. The maximum values in the PKU cohort further highlight the variability and intensity of pharmacotherapy: in five of the ten years observed, individual maximum values exceeded €20,000, peaking at €85,899 in 2020. In the matched group, while extreme values were also present (e.g. €53,479 in 2021 or €39,882 in 2014), such peaks were less frequent and occurred in the context of generally lower average utilisation.

### Rehabilitation in PKU patients

PKU patients demonstrated a higher utilisation of rehabilitation services compared to the matched control population, particularly in childhood and early adulthood. The largest difference was observed in the 2–12 years age group, with 5.6% of PKU patients receiving rehabilitation versus 0.3% among controls. Elevated rates were also found in adolescents aged 13–15 years (0.7% vs. 0.2%) and 16–17 years (1.2% vs. 0.0%). Notably, in adults aged 25–54 years, 2.3% of PKU patients underwent rehabilitation compared to 1.0% in the control group.

The most frequent reasons for rehabilitation in patients with PKU varied by developmental stage across age groups. In children aged 2–12 years, rehabilitation was commonly associated with neurodevelopmental disorders, including specific developmental disorders of speech and language (ICD-10: F80), combined developmental disorders (ICD-10: F83), pervasive developmental disorders (ICD-10: F84), and other specified developmental delays (ICD-10: F89, F82). In adolescents, frequent causes included speech or language disorders (ICD-10: R47) and behavioural problems such as conduct disorders (ICD-10: F91) (Table 1).

**Table 1:** Presents the most common diagnoses associated with rehabilitation cases, based on three-character ICD-10 codes recorded in the same case ID as the rehabilitation measure. For each age group, the 10 most frequent diagnoses are shown; ties are included where case numbers are equal

Age Group	Diagnosis	Description	Share (SHI %)
Minor	F80	Specific developmental disorders of speech and language	
	F83	Mixed specific developmental disorders	
	E10	Type 1 diabetes mellitus	
	F82	Specific developmental disorder of motor function	
	F84	Pervasive developmental disorders	
	F89	Unspecified developmental disorder	
	G81	Hemiparesis and hemiplegia	
	H52	Disorders of accommodation and refraction	
	I61	Intracerebral haemorrhage	
	J45	Bronchial asthma	
	H91	Other hearing loss	
	Q21	Congenital malformations of cardiac septa	
Adult	I10	Essential (primary) hypertension	
	M16	Coxarthrosis [arthrosis of the hip joint]	
	M54	Back pain	
	F32	Depressive episode	
	M17	Gonarthrosis [arthrosis of the knee joint]	
	I25	Chronic ischaemic heart disease	
	M51	Other intervertebral disc disorders	
	I63	Cerebral infarction	
	F43	Reactions to severe stress and adjustment disorders	

## Discussion

This analysis of real-world claims data provides a comprehensive overview of the healthcare landscape for patients with PKU in Germany over the past decade. The age-stratified approach revealed distinct patterns in comorbidity profiles, treatment utilisation, and healthcare service use across the lifespan. Our findings offer robust prevalence data, reinforce the high burden of neuropsychiatric comorbidities in PKU, and point towards critical care needs in the context of pregnancy. In addition, we observed a progressive decline in claims-documented blood Phe monitoring, dietary interventions, and pharmacological therapy with increasing age pointing to pronounced age-related reductions in claims-documented PKU care that warrant further attention in the context of lifelong management.

The prevalence of PKU estimated for the German SHI population aligns closely with previously published German data, supporting the epidemiological plausibility of our findings and, together with prior evaluations of the DADB, the suitability of the database for this analysis. A 2015 German study reported an adult PKU prevalence of 10.13 per 100,000 individuals [3]. This is well in line with the five-year prevalence estimate of 0.010% observed in our analysis, although our estimate covered individuals across the full age spectrum. Given the rarity of PKU, prevalence estimates based on observed cases in claims data are inherently subject to statistical uncertainty when extrapolated to the German SHI population. To transparently reflect this uncertainty, annual extrapolated case counts were reported with corresponding 95% confidence intervals. In addition, the five-year cumulative prevalence was used to provide a more stable period-based estimate of PKU occurrence across the observation window. While the inherent limitations of claims data and possible residual selection effects cannot be fully excluded, the close alignment with published prevalence estimates strengthens confidence in the validity of the DADB-based prevalence assessment and supports the applicability of the broader findings to routine care within the German SHI setting. Furthermore, this analysis identified a slight female predominance in the adult PKU population, also noted by [3]. This may reflect practices of intensified follow-up in women of childbearing age to prevent MPKUS [2]. At the same time, our findings raise important questions regarding the claims-documented management of PKU during pregnancy. In several cases, the diagnosis was not documented in the same year as the pregnancy, which may reflect gaps in documentation or coordination between patients and gynaecologic care providers. In addition, less than half of the affected women had claims-documented nutritional therapy during this high-risk phase, in which inadequate Phe control can result in severe and irreversible foetal harm, including microcephaly, congenital malformations, and cognitive impairment [20, 22].

Pregnancy terminations were documented in a notable proportion of women with recorded pregnancy in our cohort. Given the small number of observed cases and the absence of clinical context regarding individual pregnancy courses, this finding should be interpreted cautiously. Nevertheless, in conjunction with the limited documentation of nutritional therapy during pregnancy, it points to the particular vulnerability of this care situation and underscores the clinical relevance of maternal PKU management. Structured preconception care, consistent documentation practices, and closer integration of metabolic and gynaecologic services are urgently needed to ensure maternal and foetal safety, as emphasised by recent efforts to implement dedicated prenatal care programmes in German metabolic centres [37].

In our study, somatic comorbidities were prominently elevated in our PKU cohort, spanning multiple organ systems. This clinical profile is a key finding with important clinical implications. We found higher rates of disorders, such as osteoporosis and other bone-related disorders, gastrointestinal complaints, adiposity, hypertension, bronchial asthma, ocular and dermal complications. These findings are consistent with studies reporting increased rates of somatic conditions among individuals with PKU [3, 16, 38–40]. Collectively, this evidence supports the characterisation of PKU as a multisystemic disorder, with adults experiencing a greater overall burden of somatic comorbidities compared to unaffected peers [41].

Our real-world study confirms that PKU is associated with a high prevalence of neuropsychiatric disorders, especially mental and behavioural, which aligns with previous evidence [2, 31, 42–45]. Most notably, intellectual disability was strongly overrepresented, accompanied by increased odds for mood, depressive, hyperkinetic, anxiety, adjustment, and other behavioural disorders. Prior studies have reported similar trends, for example [31], found an almost eight-fold higher prevalence of intellectual disability and elevated rates of autism and other neuropsychiatric conditions in adults with PKU. We likewise observed an excess of affective disorders (e.g. depression and anxiety) among PKU patients, concordant with prior data showing major depression is about 2–3 times more frequent in PKU adults than in peers [3]. Hereby, the patterns show a shift with age, with cognitive impairments more prominent in childhood and adolescence, and affective and behavioural disorders increasingly observed in adulthood. These convergent findings underscore that neurocognitive and psychiatric challenges remain a relevant component of PKU across the lifespan, potentially reflecting the long-term metabolic and psychosocial burden of the disease described in previous literature.

Our findings support the notion that PKU is a multisystem disorder across the lifespan and reinforce recommendations that lifelong metabolic control may help prevent or mitigate complications [41]. Routine screening for common comorbidities should therefore be an integral part of long-term PKU management to enable timely detection and intervention.

The utilisation of nutrition therapy emerged as a critical issue. The data showed a moderate increase in the overall prescription of medical nutrition therapy over the observation period, which may initially be viewed as a positive trend. A more differentiated analysis revealed pronounced age-related disparities. While documented nutritional therapy was more common during childhood and adolescence, it was recorded for only about half of paediatric patients and declined further from early adulthood onwards, particularly beyond the age of 35 years. These findings should be interpreted in light of the limitations of claims data, as the present analysis captures reimbursed nutritional therapy based on ATC and EBM codes and may therefore underestimate the full extent of dietary management in routine care. This is particularly relevant because not all PKU-related dietary products and support services are necessarily reflected in SHI claims data. Nevertheless, the observed age-related decline is consistent with prior literature describing reduced long-term dietary management in adulthood, despite international recommendations for lifelong treatment [1, 2, 45]. Studies have documented that dietary adherence deteriorates with age, especially during the transition from adolescence to adulthood [1, 46]. The transition from paediatric to adult care appears to be a particularly vulnerable period, during which many patients lose access to structured metabolic care, face reduced reimbursement for medical and low-protein foods, or deprioritise dietary control due to psychosocial or practical challenges [8, 46, 47].

In summary, the low level of documented nutritional therapy among patients with PKU, particularly in adulthood, should not be interpreted as a complete measure of dietary management. However, together with the pronounced age-related decline and prior evidence on challenges in maintaining lifelong dietary treatment, these findings point to a persistent need to strengthen age-adapted nutritional support in PKU care. In the German healthcare setting, the comparatively low level of claims-documented nutritional therapy also raises the question of whether dietary management in a severe, lifelong condition such as PKU is sufficiently and continuously reflected and supported within existing care and reimbursement structures. Efforts should therefore be intensified to ensure that individuals with PKU are successfully transitioned into lifelong dietary management and receive adequate support throughout the course of the disease. This includes, but is not limited to, improved reimbursement policies for adults, dedicated age-adapted metabolic care structures for lifelong management, and expanded access to nutritional counselling and support services [48]. Therefore, reinforcing the “diet for life” principle must become a central goal in PKU healthcare delivery and policy planning.

In this context, pharmacological therapies for PKU, namely sapropterin dihydrochloride and pegvaliase, represent important adjuncts but were documented only in a minority of patients in routine care. In our dataset, fewer than 20% of patients received any pharmacological therapy during the observation period, with utilisation rates declining markedly with increasing age. Sapropterin dihydrochloride accounted for approximately 10% of the documented pharmacological treatment. Of note, no pharmacologic treatment was documented in patients above 55 years of age. Compared to earlier data, this still reflects a gradual increase in adoption. For example, a 2015 German claims analysis reported sapropterin dihydrochloride use in fewer than 1% of adult PKU patients [3]. However, the interpretation of these treatment rates requires particular caution. Claims data do not provide information on PKU phenotype, residual PAH activity, or BH4 responsiveness, all of which are relevant for treatment eligibility. The limited prescription rate of sapropterin dihydrochloride observed in our study must be interpreted in light of its restricted biological applicability, as meaningful Phe reductions are achieved only in a subset of patients with residual PAH activity [45, 49]. Accordingly, low population-level treatment rates should not be interpreted as direct evidence of insufficient pharmacological care.

Another pharmacological option for patients with PKU is pegvaliase, an enzyme substitution therapy approved in Europe since 2019. In our cohort, however, fewer than ten patients received a prescription for pegvaliase, indicating its limited documented use during the observation period. Clinical trial data confirm that pegvaliase can lead to substantial and sustained reductions in blood Phe levels [50]. However, its use is limited by practical challenges such as extended titration phases, complex dosing regimens, and a demanding immunological profile [13, 51–53]. Adverse events like anaphylaxis and hypersensitivity also require close monitoring and the implementation of risk mitigation strategies. Despite these barriers, existing evidence suggests that pegvaliase can be successfully implemented and maintained in adult patients if supported by careful patient selection, appropriate education, and multidisciplinary care structures [54].

In summary, pharmacological therapies were documented only in a minority of patients with PKU in routine care, indicating limited population-level use during the observation period. At the same time, these treatment rates should not, on their own, be interpreted as evidence of insufficient pharmacological care, as claims data do not allow treatment use to be assessed against individual clinical eligibility. Nevertheless, the findings provide relevant real-world evidence on the current uptake of pharmacological options in long-term PKU management. In this context, novel treatment approaches such as sepiapterin (PTC923, Sephience™) represent a promising advance [49]. Recent clinical trial data support its therapeutic potential, showing sustained reductions in blood Phe levels alongside improved dietary flexibility [55]. These characteristics position sepiapterin as a relevant emerging treatment option in the evolving therapeutic landscape of PKU. Further evidence from routine clinical practice will be needed to assess its role in long-term care.

As a further aspect of PKU management, blood Phe monitoring remains a clinical cornerstone, enabling timely assessment of metabolic control and therapy adjustments. Clinical guidelines consistently recommend regular blood testing across the lifespan to ensure Phe levels remain within therapeutic targets [2, 45, 56]. It is essential to acknowledge the structural limitations inherent in claims data. In Germany, as well as in other countries around the world, a considerable share of PKU care is provided by SPZ and AMBO [7, 57–59]. These institutions bill services outside of the routine SHI system, and many of their activities, such as Phe testing or dietary counselling, are not captured in our dataset. As a result, relevant aspects of disease monitoring remain underrepresented in our dataset.

Despite this lack of documentation, our data still show substantial patient engagement within these specialised settings during childhood and adolescence. At the same time, the use of SPZ and AMBO care decreased markedly with increasing age, accompanied by a pronounced decline in claims-documented Phe blood testing. While the available data do not allow a clear distinction between underdocumentation of monitoring and a true reduction in testing frequency, the parallel decline in specialised care utilisation and documented monitoring in adulthood points to a potentially relevant transition-related challenge in long-term PKU follow-up. Given the well-established link between sustained Phe control and favourable long-term cognitive and physical outcomes, this pattern warrants particular attention. It underscores the importance of robust, lifelong monitoring structures that support continuous biochemical surveillance and individualised therapy adjustments across all age groups.

The cost analyses were primarily intended to provide a comparative description of healthcare expenditures in patients with PKU and the matched control population. To contextualise the observed year-to-year variability, potential explanatory factors were examined, including the introduction of new PKU-specific treatment options and other developments relevant to cost patterns. However, no distinct or consistent temporal drivers were identified. Likewise, no changes in the German healthcare or reimbursement system were apparent that would specifically explain the observed fluctuations. Individual high-cost cases may have contributed to pronounced peaks in selected years, but these could not be further disaggregated due to data protection requirements. Overall, the cost findings therefore primarily reflect the higher medical resource use associated with PKU rather than identifiable year-specific cost drivers. From a clinical and health-economic perspective, strengthening age-appropriate monitoring and metabolic control may have implications that extend beyond short-term biochemical targets. Better and sustained control may reduce the incidence and severity of neuropsychiatric, skeletal and cardiometabolic comorbidities such as depression and anxiety, osteoporosis, obesity, hypertension and diabetes, which are additional major drivers of healthcare utilisation and costs in PKU. Although causality cannot be inferred from claims data, the convergence of evidence linking poor control to adverse long-term outcomes supports this rationale. Improvements in lifelong, coordinated care could therefore translate into fewer outpatient and inpatient encounters, lower pharmaceutical spending for comorbidity management, and a reduction in indirect burdens such as productivity losses and caregiver time. Overall, the findings point to important challenges in the lifelong management of PKU and underscore the need for coordinate, age-appropriate care strategies, with particular focus on transitional phases and sustained metabolic monitoring.

### Limitations

This study is based on administrative healthcare claims data, which offer valuable insights into the utilisation of healthcare services but are associated with certain limitations. As the data are primarily collected for reimbursement purposes, clinical information such as Phe concentrations, genotypic characteristics, treatment-relevant phenotypic information such as BH4 responsiveness, dietary adherence and psychosocial burden, which may influence healthcare utilisation and outcomes, are not captured in claims data. In addition, services provided in specialised settings, such as university hospitals or social paediatric centres, may be reimbursed outside the standard accounting system and are therefore not fully available. As the German healthcare system does not integrate caregiver burden and indirect costs, this study offers robust estimates of healthcare service utilisation and direct medical costs. It does not capture the full societal impact of PKU. Patients without a documented diagnosis or those no longer in active care could not be captured, as individuals are only visible in claims data when interacting with the healthcare system, despite continuous insurance coverage. This may limit the completeness of the overall picture. This may also result in residual confounding by factors not fully observable in claims data, including socioeconomic characteristics. Furthermore, the comorbidity analysis was exploratory in nature and involved the evaluation of a broad range of diagnostic categories. No adjustment for multiple comparisons was applied. Accordingly, individual statistically significant associations should be interpreted as exploratory findings rather than confirmatory evidence. Diagnosis coding based on the ICD-10 classification (e.g. E70.0 and E70.1) allows differentiation between classical PKU and other hyperphenylalaninemias at a broad diagnostic level but does not provide sufficient granularity regarding disease severity or detailed phenotypic manifestations within the PKU spectrum. The broader claims-based case definition was chosen deliberately to also capture milder phenotypic manifestations that remain relevant from a healthcare services research perspective. Consequently, the study population may have included individuals with varying clinical severity, and some degree of diagnostic heterogeneity and potential misclassification cannot be excluded. The inclusion of milder phenotypic manifestations may therefore have influenced estimates of disease burden, healthcare utilisation, and treatment intensity among patients with more severe classical PKU. Due to the rarity of the disease, some subgroup analyses were limited by small case numbers, and reporting was subject to data protection requirements. Extrapolated prevalence figures should be understood as estimates of the expected prevalence range within the German SHI population rather than as directly observed national case counts. To reflect the statistical uncertainty associated with prevalence estimation in a rare disease setting, 95% confidence intervals were calculated for annual extrapolated case numbers. In addition, the five-year cumulative prevalence was reported to provide a more stable period-based estimate and to mitigate the influence of annual fluctuations in observed case numbers.

At the same time, the study has several important strengths. It draws on the DADB, a large longitudinal SHI claims database that, in the present analysis, comprised approximately 2.6 to 3.0 million continuously insured individuals per reporting year. Given the nearly complete coverage of reimbursed healthcare services and the very limited patient cost sharing in the German SHI system, the data provide a broad and consistent picture of documented care and service utilisation. The large underlying population enabled the identification of a meaningful number of patients with PKU and allowed for age-stratified and treatment-specific analyses. To our knowledge, this study represents one of the most comprehensive assessments of PKU care in Germany based on real-world data.

## Conclusion

This real-world study highlights persistent challenges in the lifelong management of PKU in Germany, including low levels of documented pharmacological treatment, declining care continuity in adulthood and a high burden of neuropsychiatric and somatic comorbidities. These findings reinforce the need for integrated, guideline-concordant care across all age groups.

Our findings call for renewed efforts to improve PKU care structures in Germany, to close known gaps, support long-term adherence and deliver care that not only preserves metabolic control but also respects the lived realities of patients. Rare diseases like PKU must not disappear from the care agenda once childhood is over, they require sustained attention, investment and compassion across the lifespan.

## Supplementary Information

Below is the link to the electronic supplementary material.

Supplementary Material 1

## Data Availability

The data used in this study cannot be made available in the manuscript, the supplemental files, or in a public repository due to German data protection laws (Bundesdatenschutzgesetz). Therefore, they are stored on a secure drive at the Gesundheitsforen Leipzig GmbH to facilitate replication of the results. Generally, access to data of sick funds for research purposes is possible only under the conditions defined in German Social Law (SGB V § 287). Access to the data used in this study can only be provided to external parties under the conditions of the cooperation contract of this research project and after written approval by the Gesundheitsforen Leipzig GmbH.
