# Supplementary material for: Care of patients with Phenylketonuria (PKU) in Germany – a claims data analysis from 2013 to 2023
Source: Orphanet J Rare Dis. 2026 Jul 2;21:237. doi: 10.1186/s13023-026-04467-3 (PMC13332590; doi:10.1186/s13023-026-04467-3)
Supplement: Supplementary file 1 — Supplementary Material 1 [file 13023_2026_4467_MOESM1_ESM.docx]

# **Supplement**

**Table S1** Most frequent comorbidities based on odds ratios in the PKU population compared to a matched cohort. Comorbidity is included if the odds ratio is statistically significant> 1. Odds ratios [±95% confidence interval] from the conditional logistic regression.

| **ICD-10 code** | **Description** | **Odds ratio (95% CI)** | **P-value** |
| --- | --- | --- | --- |
| E70 | Disorders of aromatic amino-acid metabolism | 3251310269138.63 (0; Inf) | 0.997 |
| F79 | Unspecified mental retardation | 16.07 (11.78; 21.92) | 0.000 |
| E88 | Other metabolic disorders | 9.19 (7.44; 11.36) | 0.000 |
| M81 | Osteoporosis without pathological fracture | 3.40 (2.57; 4.51) | 0.000 |
| Z01 | Other special examinations and investigations of persons without complaint or reported diagnosis | 3.00 (2.67; 3.37) | 0.000 |
| R45 | Symptoms and signs involving emotional state | 2.74 (2.14; 3.50) | 0.000 |
| R32 | Unspecified urinary incontinence | 2.61 (2.00; 3.41) | 0.000 |
| F90 | Hyperkinetic disorders | 2.12 (1.66; 2.71) | 0.000 |
| K59 | Other functional intestinal disorders | 1.89 (1.55; 2.31) | 0.000 |
| R29 | Other symptoms and signs involving the nervous and musculoskeletal systems | 1.85 (1.44; 2.36) | 0.000 |
| K29 | Gastritis and duodenitis | 1.68 (1.40; 2.02) | 0.000 |
| F98 | Other behavioural and emotional disorders with onset usually occurring in childhood and adolescence | 1.63 (1.26; 2.10) | 0.000 |
| B37 | Candidiasis | 1.61 (1.26; 2.05) | 0.000 |
| H10 | Conjunctivitis | 1.56 (1.34; 1.81) | 0.000 |
| F33 | Recurrent depressive disorder | 1.55 (1.22; 1.97) | 0.000 |
| F43 | Reaction to severe stress, and adjustment disorders | 1.54 (1.31; 1.81) | 0.000 |
| E79 | Disorders of purine and pyrimidine metabolism | 1.52 (1.17; 1.97) | 0.001 |
| K21 | Gastro-oesophageal reflux disease | 1.48 (1.21; 1.81) | 0.000 |
| Z25 | Need for immunization against other single viral diseases | 1.47 (1.29; 1.68) | 0.000 |
| L20 | Atopic dermatitis | 1.45 (1.24; 1.71) | 0.000 |
| Z71 | Persons encountering health services for other counselling and medical advice, not elsewhere classified | 1.44 (1.22; 1.70) | 0.000 |
| E66 | Obesity | 1.40 (1.20; 1.63) | 0.000 |
| T78 | Adverse effects, not elsewhere classified | 1.40 (1.18; 1.66) | 0.000 |
| I10 | Essential (primary) hypertension | 1.39 (1.19; 1.64) | 0.000 |
| M53 | Other dorsopathies, not elsewhere classified | 1.38 (1.14; 1.66) | 0.001 |
| J45 | Asthma | 1.30 (1.11; 1.52) | 0.001 |
| Z76 | Persons encountering health services in other circumstances | 1.29 (1.00; 1.68) | 0.049 |
| N94 | Pain and other conditions associated with female genital organs and menstrual cycle | 1.29 (1.05; 1.59) | 0.014 |
| M21 | Other acquired deformities of limbs | 1.29 (1.06; 1.56) | 0.008 |
| K52 | Other noninfective gastroenteritis and colitis | 1.29 (1.04; 1.59) | 0.016 |
| M62 | Other disorders of muscle | 1.28 (1.04; 1.59) | 0.020 |
| M41 | Scoliosis | 1.27 (1.01; 1.60) | 0.036 |
| R52 | Pain, not elsewhere classified | 1.25 (1.00; 1.56) | 0.042 |
| F41 | Other anxiety disorders | 1.24 (1.01; 1.54) | 0.039 |
| H61 | Other disorders of external ear | 1.24 (1.03; 1.49) | 0.021 |
| M47 | Spondylosis | 1.24 (1.00; 1.52) | 0.041 |
| F45 | Somatoform disorders | 1.23 (1.05; 1.45) | 0.010 |
| H52 | Disorders of refraction and accommodation | 1.17 (1.05; 1.32) | 0.005 |

**Table S2** Annual total healthcare costs for incident PKU patients in Germany (2014-2023). For each year, the number of incident PKU patients, minimum, mean, median, and maximum total costs are shown in euros (€).

| **Reporting year** | **n persons in population, DADB; SHI** | **Total costs, minimum €** | **Total costs,**  **mean €** | **Total costs, median €** | **Total costs, maximum €** |
| --- | --- | --- | --- | --- | --- |
| 2014 | 33; 927 | 93 | 3,556 | 1,692 | 24,661 |
| 2015 | 31; 909 | 49 | 5,219 | 1,165 | 56,170 |
| 2016 | 24; 648 | 86 | 3,254 | 1,595 | 14,587 |
| 2017 | 31; 790 | 68 | 9,264 | 2,000 | 104,770 |
| 2018 | 33; 888 | 157 | 6,528 | 1,599 | 28,211 |
| 2019 | 20; 499 | 42 | 10,538 | 4,400 | 49,453 |
| 2020 | 33; 871 | 31 | 20,485 | 7,580 | 281,691 |
| 2021 | 21; 521 | 60 | 4,227 | 1,206 | 25,962 |
| 2022 | 19; 522 | 73 | 4,817 | 2,380 | 29,939 |
| 2023 | 19; 494 | 340 | 7,067 | 1,691 | 56,029 |

**Table S3** Annual total healthcare costs for the matching control population (2014-2023). This table presents total annual healthcare costs per person for the matched control cohort without PKU, derived from the DADB and extrapolated to the statutory health insurance (SHI) population. For each year, the number of individuals, minimum, mean, median, and maximum total costs are shown in euros (€).

| **Reporting year** | **n persons in matched cohort,**  **DADB; SHI** | **Total costs, minimum €** | **Total costs,**  **mean €** | **Total costs, median €** | **Total costs, maximum €** |
| --- | --- | --- | --- | --- | --- |
| 2014 | 330; 9,270 | 0 | 2,431 | 731 | 101,999 |
| 2015 | 310; 9,085 | 0 | 2,258 | 726 | 43,143 |
| 2016 | 240; 6,477 | 0 | 1,896 | 661 | 27,904 |
| 2017 | 310; 7,899 | 0 | 1,817 | 632 | 37,781 |
| 2018 | 330; 8,878 | 0 | 2,049 | 700 | 43,448 |
| 2019 | 200; 4,992 | 0 | 2,374 | 750 | 34,756 |
| 2020 | 330; 8,708 | -354 | 1,851 | 566 | 44,697 |
| 2021 | 210; 5,208 | 0 | 2,655 | 656 | 61,102 |
| 2022 | 190; 5,222 | 0 | 2,723 | 825 | 61,142 |
| 2023 | 190; 4,937 | 0 | 3,247 | 770 | 155,229 |

**Table S4** Annual inpatient healthcare costs among patients with incident PKU (2014–2023). This table shows the inpatient component of total annual healthcare costs for individuals with incident PKU, derived from the DADB and extrapolated to the SHI population. Reported are the number of individuals and their corresponding minimum, mean, median, and maximum inpatient costs in euros (€) for each reporting year.

| **Reporting year** | **n persons in population, DADB; SHI** | **Total costs, minimum €** | **Total costs,**  **mean €** | **Total costs, median €** | **Total costs, maximum €** |
| --- | --- | --- | --- | --- | --- |
| 2014 | 33; 927 | 0 | 1,217 | 0 | 12,646 |
| 2015 | 31; 909 | 0 | 2,559 | 0 | 49,062 |
| 2016 | 24; 648 | 0 | 1,236 | 0 | 6,572 |
| 2017 | 31; 790 | 0 | 5,361 | 0 | 90,665 |
| 2018 | 33; 888 | 0 | 2,826 | 0 | 22,537 |
| 2019 | 20; 499 | 0 | 4,079 | 0 | 17,373 |
| 2020 | 33; 871 | 0 | 12,087 | 2,082 | 249,186 |
| 2021 | 21; 521 | 0 | 1,131 | 0 | 5,635 |
| 2022 | 19; 522 | 0 | 1,090 | 0 | 13,557 |
| 2023 | 19; 494 | 0 | 4,786 | 0 | 50,914 |

**Table S5** Annual inpatient healthcare costs in the matched control population (2014–2023). This table provides inpatient cost data for the matched control group without PKU, based on DADB data extrapolated to the SHI population. For each year, the number of individuals, minimum, mean, median, and maximum inpatient costs in euros (€) are listed.

| **Reporting year** | **n persons in matched cohort,**  **DADB; SHI** | **Total costs, minimum €** | **Total costs,**  **mean €** | **Total costs, median €** | **Total costs, maximum €** |
| --- | --- | --- | --- | --- | --- |
| 2014 | 330; 9,270 | 0 | 1,009 | 0 | 89,887 |
| 2015 | 310; 9,085 | 0 | 877 | 0 | 27,712 |
| 2016 | 240; 6,477 | 0 | 674 | 0 | 18,600 |
| 2017 | 310; 7,899 | 0 | 768 | 0 | 35,562 |
| 2018 | 330; 8,878 | 0 | 745 | 0 | 35,310 |
| 2019 | 200; 4,992 | 0 | 1,079 | 0 | 33,930 |
| 2020 | 330; 8,708 | -354 | 868 | 0 | 41,512 |
| 2021 | 210; 5,208 | 0 | 780 | 0 | 35,665 |
| 2022 | 190; 5,222 | 0 | 925 | 0 | 37,412 |
| 2023 | 190; 4,937 | 0 | 1,873 | 0 | 150,315 |

**Table S6** Annual outpatient healthcare costs among patients with incident PKU (2014–2023). This table summarises outpatient healthcare costs for individuals with incident PKU based on DADB data extrapolated to the German SHI population. It includes the number of patients per year, along with minimum, mean, median, and maximum outpatient costs in euros (€).

| **Reporting year** | **n persons in population, DADB; SHI** | **Total costs, minimum €** | **Total costs,**  **mean €** | **Total costs, median €** | **Total costs, maximum €** |
| --- | --- | --- | --- | --- | --- |
| 2014 | 33; 927 | 58 | 911 | 536 | 6,264 |
| 2015 | 31; 909 | 49 | 953 | 577 | 3,240 |
| 2016 | 24; 648 | 71 | 745 | 551 | 2,652 |
| 2017 | 31; 790 | 33 | 1,159 | 781 | 4,926 |
| 2018 | 33; 888 | 97 | 1,075 | 796 | 5,913 |
| 2019 | 20; 499 | 42 | 1,226 | 677 | 4,616 |
| 2020 | 33; 871 | 31 | 1,819 | 1,046 | 17,201 |
| 2021 | 21; 521 | 50 | 802 | 526 | 2,608 |
| 2022 | 19; 522 | 66 | 1,234 | 973 | 4,422 |
| 2023 | 19; 494 | 104 | 1,127 | 578 | 3,081 |

**Table S7** Annual outpatient healthcare costs in the matched control population (2014–2023). Outpatient healthcare costs for the matched control group without PKU are shown, based on extrapolated DADB data. For each year, the table provides the number of individuals and corresponding minimum, mean, median, and maximum outpatient costs in euros (€).

| **Reporting year** | **n persons in matched cohort,**  **DADB; SHI** | **Total costs, minimum €** | **Total costs,**  **mean €** | **Total costs, median €** | **Total costs, maximum €** |
| --- | --- | --- | --- | --- | --- |
| 2014 | 330; 9,270 | 0 | 458 | 325 | 5,373 |
| 2015 | 310; 9,085 | 0 | 499 | 337 | 5,204 |
| 2016 | 240; 6,477 | 0 | 459 | 334 | 2,677 |
| 2017 | 310; 7,899 | 0 | 481 | 309 | 7,089 |
| 2018 | 330; 8,878 | 0 | 480 | 377 | 5,588 |
| 2019 | 200; 4,992 | 0 | 509 | 351 | 5,081 |
| 2020 | 330; 8,708 | 0 | 472 | 377 | 4,050 |
| 2021 | 210; 5,208 | 0 | 549 | 360 | 6,796 |
| 2022 | 190; 5,222 | 0 | 571 | 407 | 5,523 |
| 2023 | 190; 4,937 | 0 | 591 | 396 | 5,323 |

**Table S8** Annual drug-related healthcare costs among patients with incident PKU (2014–2023). This table presents the pharmaceutical expenditure for patients with incident PKU in the extrapolated SHI population. Data include yearly patient counts, and minimum, mean, median, and maximum costs per individual in euros (€).

| **Reporting year** | **n persons in population, DADB; SHI** | **Total costs, minimum €** | **Total costs,**  **mean €** | **Total costs, median €** | **Total costs, maximum €** |
| --- | --- | --- | --- | --- | --- |
| 2014 | 33; 927 | 0 | 679 | 115 | 5,362 |
| 2015 | 31; 909 | 0 | 1,090 | 219 | 11,352 |
| 2016 | 24; 648 | 0 | 1,094 | 167 | 8,080 |
| 2017 | 31; 790 | 0 | 1,987 | 71 | 20,388 |
| 2018 | 33; 888 | 0 | 1,262 | 109 | 16,946 |
| 2019 | 20; 499 | 0 | 3,203 | 138 | 44,166 |
| 2020 | 33; 871 | 0 | 5,840 | 167 | 85,899 |
| 2021 | 21; 521 | 0 | 1,428 | 95 | 18,708 |
| 2022 | 19; 522 | 0 | 1,027 | 663 | 7,150 |
| 2023 | 19; 494 | 0 | 676 | 24 | 8,417 |

**Table S9** Annual drug-related healthcare costs in the matched control population (2014–2023). Drug-related healthcare costs are reported for the matched control cohort without PKU, based on DADB data extrapolated to the SHI population. The table includes the number of individuals and associated minimum, mean, median, and maximum costs per year in euros (€).

| **Reporting year** | **n persons in matched cohort,**  **DADB; SHI** | **Total costs, minimum €** | **Total costs,**  **mean €** | **Total costs, median €** | **Total costs, maximum €** |
| --- | --- | --- | --- | --- | --- |
| 2014 | 330; 9,270 | 0 | 374 | 41 | 39,882 |
| 2015 | 310; 9,085 | 0 | 364 | 50 | 23,421 |
| 2016 | 240; 6,477 | 0 | 356 | 41 | 20,264 |
| 2017 | 310; 7,899 | 0 | 172 | 32 | 4,680 |
| 2018 | 330; 8,878 | 0 | 410 | 46 | 21,735 |
| 2019 | 200; 4,992 | 0 | 344 | 39 | 27,949 |
| 2020 | 330; 8,708 | 0 | 225 | 24 | 12,171 |
| 2021 | 210; 5,208 | 0 | 593 | 35 | 53,479 |
| 2022 | 190; 5,222 | 0 | 442 | 59 | 38,652 |
| 2023 | 190; 4,937 | 0 | 208 | 42 | 5,671 |
